# *Michanthidium almeidai*, a new species from northeastern Brazil (Hymenoptera, Megachilinae)

**DOI:** 10.3897/zookeys.132.1840

**Published:** 2011-10-03

**Authors:** Danúncia Urban, Daniele Regina Parizotto

**Affiliations:** 1 Laboratório de Biologia Comparada de Hymenoptera, Departamento de Zoologia, Universidade Federal do Paraná, Caixa Postal 19020, 81531-980, Curitiba, Paraná, Brazil

**Keywords:** Anthidiini, Apidae, Neotropical, taxonomy

## Abstract

A new species of *Michanthidium* Urban (Hymenoptera, Megachilinae)is described and figured from Sergipe and Bahia States, northeastern Brazil. An identification key, illustrations, and a distribution map for the three species of the genus are presented. The male genitalia of *Michanthidium almeidai* **sp. n.** and *Michanthidium albitarse* are illustrated and compared for the first time.

## Introduction

*Michanthidium* Urban, 1995 is an exclusively Neotropical anthidiine genus, according to the classification proposed by Urban and Moure (2007), although it is considered by Michener (2007) as a subgenus of *Hypanthidioides* Moure, 1947, along with nine other subgenera. Two species of *Michanthidium*, *Michanthidium  sakagamii* (Urban)and *Michanthidium  ferrugineum* (Urban) were originally described in *Gnathanthidium* Urban, 1993 and subsequently renamed by Urban (1995), because it was a junior homonym of *Gnathanthidium* Pasteels, 1969. In a recent paper, Gonzalez and Griswold (2011) proposed a new combination, *Hypanthidioides (Michanthidium) albitarsis* (Friese, 1916)(here considered *Michanthidium albitarse*) as the oldest name, resulting in *Michanthidium sakagamii* as a junior synonym.

The species of the genus can be recognized by the following combination of characters present in both sexes: presence of a juxtantennal carina; strong hooked hairs on the underside of the labial palpus and throughout the length of the galeal blade; omaulus with short carina dorsally; pronotal lobe with curved lamellae in profile view; and base of propodeum with row of lateral pits. Additionally, males are characterized by the basal tooth of mandibles separated from the middle tooth by a broad concave margin; fifth sternum with lateral spine-like projections and the presence of arolia. The females are distinguished by the mandibles with smooth apical margin between two small apical teeth and the basal angle, and the external surface with slender carinae not reaching basal half of mandible.

**Figure 1.**
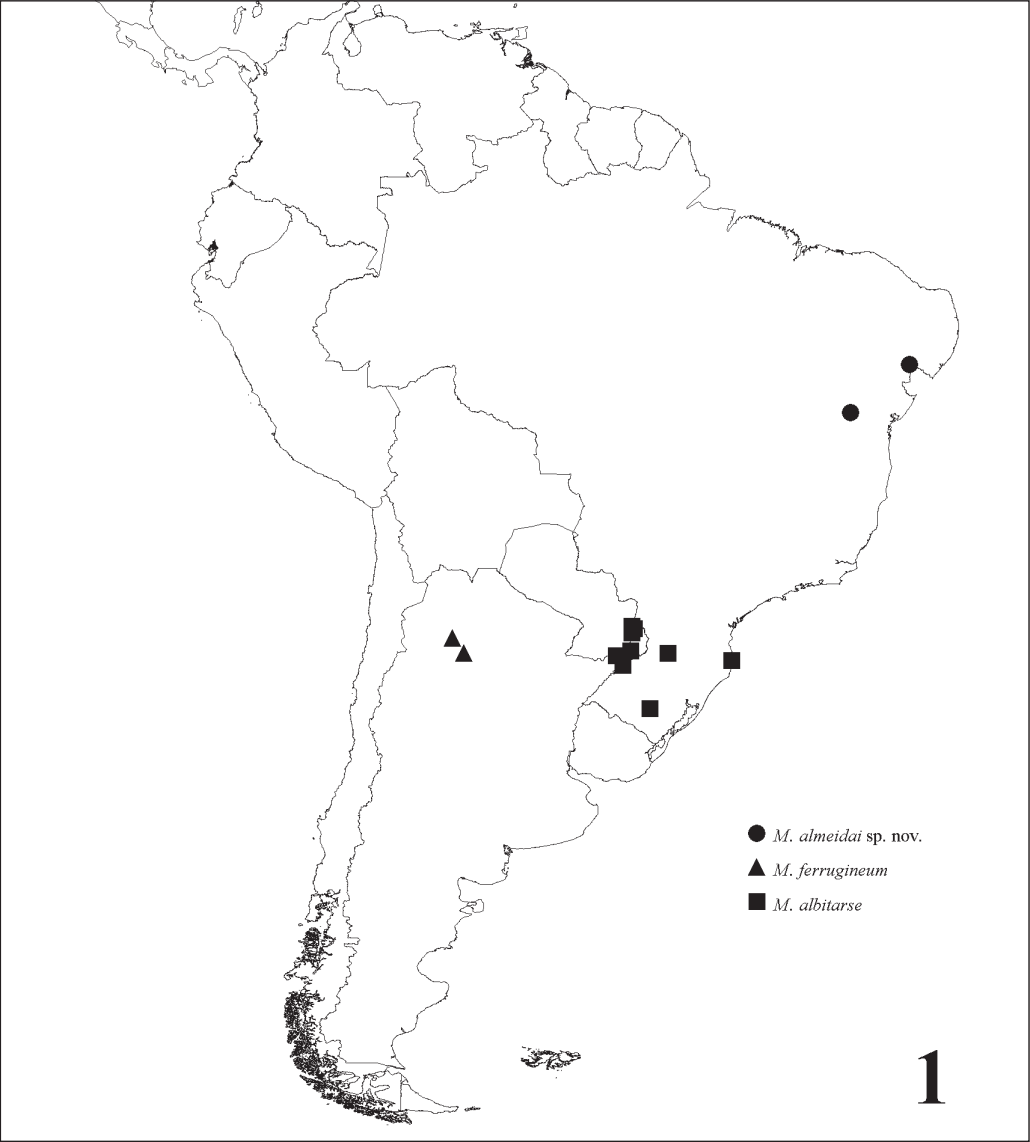
Collecting record map of species of *Michanthidium*.

The species of this genus were known from southern Brazil and Argentina, with *Michanthidium ferrugineum* (Urban, 1993) from Argentina (Tucuman) and *Michanthidium albitarse* (Friese, 1916) from Brazil (Paraná, Santa Catarina and Rio Grande do Sul) and Argentina (Misiones). The new species, described here is the first record of the genus from northeastern Brazil (Fig. 1). The key presented here is based on the material we had on hands of the three species, except for the male of *Michanthidium ferrugineum*, included here based on the description provided by Gonzalez and Griswold (2011). The map here included was based only on the material examined by us and by Gonzalez and Griswold (2011), except by the type of *Michanthidium  albitarse* that has a label from Costa Rica and it is probably an error as suggested by those authors.

## Material and methods

The measurements are given in millimeters. T and S are used here for metasomal terga and metasomal sterna, respectively. Total length was measured in lateral view, from head to apex of metasoma; length of forewing was measured at anterior margin, from the costal sclerite to the wing apex. The type material is deposited in the following institutions: Coleção Entomológica Pe. Jesus Santiago Moure, Departamento de Zoologia, Universidade Federal do Paraná, Curitiba, Brazil (DZUP) and Coleção Entomológica do Departamento de Zoologia, Universidade Federal de Minas Gerais, Belo Horizonte (DZMG).

## Taxonomy

### Michanthidium almeidai

Urban & Parizotto sp. n.

urn:lsid:zoobank.org:act:39581B7E-DF5E-4987-876D-A2C6B6D24B57

http://species-id.net/wiki/Michanthidium_almeidai

Figs 4–7 8, 11, 13, 17 18, 20, 22–23 

#### Diagnosis.

Tegula sparsely punctate; lateral margin of axilla subangulate; posterior margin of scutellum weakly emarginated at middle (Fig. 11); integument black with yellow maculae. Male with light yellow bands in all terga, laterally wider; distal tergum bilobed with a rounded median emargination (Fig. 20). First to fifth terga of female with yellow bands, slightly light brown colored; bands wider laterally on T1–T3 (Fig. 13).

#### Description.

Holotype female. Length 6.22; length of forewing 4.90; width of head 2.50; length of eye 1.67; upper interorbital distance 1.55; lower interorbital distance 1.12. Integument black with the following yellow areas: paraocular maculae almost reaching upper angle of compound eyes, irregularly narrowing above antennal sockets; interalveolar maculae narrow at middle, butterfly-shaped (Fig. 4); postocellar band extending across vertex to dorsal third of gena (Fig. 6). Pronotal lobe with spot; mesoscutum with band along anterior and lateral margins; axilla and posterior margin of scutellum. Tegula amber with small spot near base. Wings dark brown, darker at base and at costal margin (Figs 6 and 8). Fore and middle legs with coxae and trochanters black; femora with light brown area and a light yellow maculae on external surface; tibiae light brown with yellow band elongated; tarsomeres light brown. Hind legs with coxae black and with a light yellow spot; trochanter black; femora light brown with yellow band narrower than in anterior femora; tibiae light brown with a narrow yellow basal band; tarsomeres darkened. T1-T5 with yellow bands, distinctly wider laterally on first basal three terga; distal tergum black.

Pilosity: color predominantly light yellow, hairs sparse, longer than ocellar diameter, on paraocular area denser and longer; posterior tibiae with dense and plumose hairs; scopa white.

Structure: mandible punctate, punctures smaller and denser on distal half, external surface with slender carinae not extending to basal half. Head and mesosoma densely punctate; posterior margin of scutellum weakly emarginated at middle; terga with punctures sparser than those of head and mesosoma, sparser and shallower on disc than on sides.

Paratype male. As in female except for denser pilosity and the following: Length 6.24; length of forewing 5.09; width of head 2.48; length of eye 1.52; upper interorbital distance 1.55; lower interorbital distance 1.01. Integument predominantly black, with the following yellow areas: mandibles, except teeth and borders; clypeus; inferior paraocular area; translucent interalveolar carinae with yellow narrow spot. Ventral surface of scape almost entirely yellow; pedicel and ventral surface of flagellomeres darkened; dorsal surface brown (Fig. 5). Fore and middle legs with coxae and trochanters black; femora and tibiae dark brown with large light brown area; femora with yellow elongated internal band; tibiae with light yellow elongated external band; basitarsus amber on external surface. Hind legs with coxae with large yellow maculae on ventral surface; femora almost entirely black, with apical half of ventral surface ferruginous, external surface with small yellow macula; tibiae almost entirely ferruginous with two yellow maculae, one basal and one apical; basitarsus and tarsomeres light brown. Basal tergum light brown; first and second terga with marginal area ferruginous; all terga with light yellow subapical bands, wider laterally on T1–T5, distinctly wider on T1; T6 band wider at middle; distal tergum almost entirely light yellow, black only at base and with translucent ferruginous margin (Figs 7, 18, 20 and 22). Sterna brown with light yellow irregular and narrow median bands.

Structure. Distal tergum medially emarginated on distal margin, thus forming two lobes with lateral margins medially convergent (Fig. 20).

Genitalia. Gonostylus as long as penis valves, about the same width across length; gonocoxites joined by narrow area with base projected. Penis valves with apodemes shorter than in *Michanthidium albitarse* (Fig. 23). The gonostylus are narrow on the apical third in *Michanthidium albitarse*, and distinctly incurved (Fig. 24).

Type material. Holotype female. BRAZIL, Sergipe. “Canindé do **[sic]** São Fran- / cisco – SE, Sta Maria / Brasil 23.09.2005 / Debora Moura leg.”, “L 172 P 140 / *Scoparia dulcis*” (DZUP). One male paratype: same data as holotype, except by “25.10.2005, “L 172 P1190 / *Schultesia guianensis”* (DZUP). One male paratype: BRAZIL, Bahia. “Lençois, BA / BRASIL 08/01/1997 / E. A. B. Almeida” (DZMG).

**Figures 2–7.**
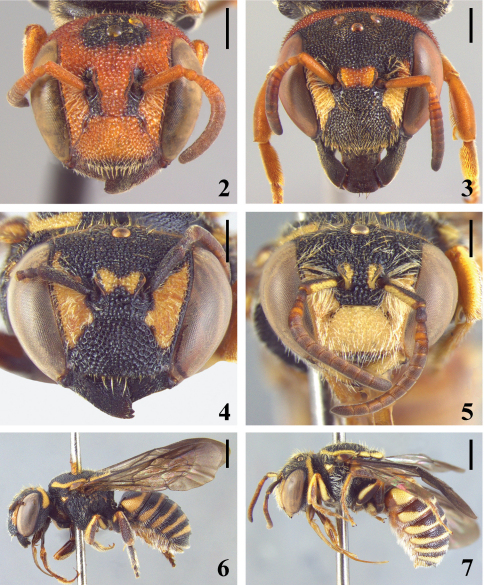
**2–4** head in frontal view of female **2** *Michanthidium  ferrugineum* **3** *Michanthidium  albitarse* **4** *Michanthidium almeidai* sp. n.  **5** head in frontal view of male of *Michanthidium  almeidai* sp. n. **6–7** lateral view **6** female of *Michanthidium almeidai* sp. n. **7** male of *Michanthidium almeidai* sp. n. Scale line = 0.5 mm (Figures 2–5). Scale line = 1.0 mm (Figures 6–7).

**Figures 8–17.**
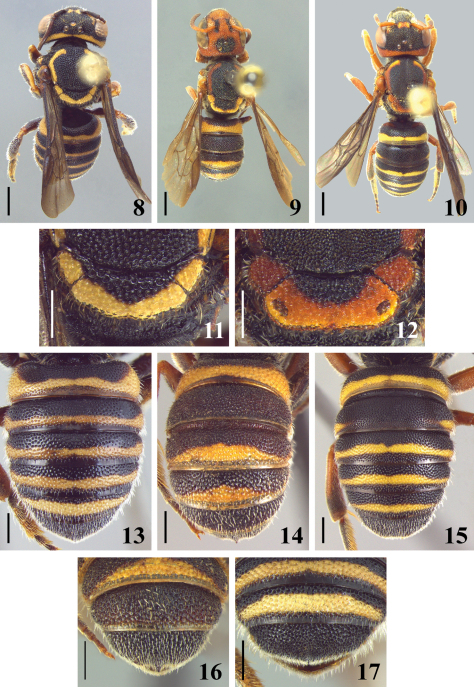
**8–10** dorsal view of female **8** *Michanthidium almeidai* sp. n. **9** *Michanthidium ferrugineum* **10** *Michanthidium albitarse* **11–12** dorsal view of scutellum and axilla **11** *Michanthidium almeidai* sp. n. **12** *Michanthidium  albitarse* **13–15** dorsal view of metasoma of female **13** *Michanthidium almeidai* sp. n. **14** *Michanthidium ferrugineum* **15** *Michanthidium albitarse* **16–17** apex of metasoma of female **16** *Michanthidium ferrugineum* **17** *Michanthidium almeidai* sp. n.

**Figures 18–24.**
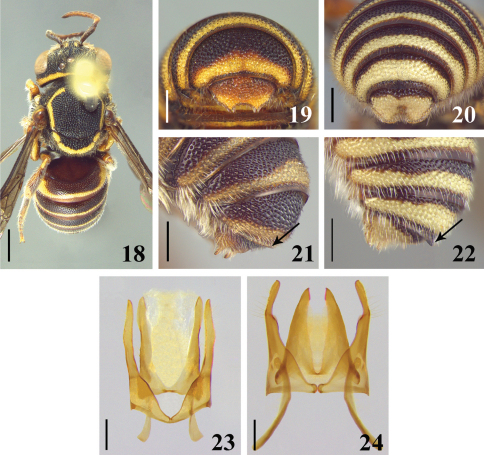
**18** dorsal view of male of *Michanthidium almeidai* sp. n. **19–20** dorsal view of apex of metasoma of male **19** *Michanthidium albitarse* **20** *Michanthidium almeidai* sp. n. **21–22** apex of T6 of male **21** *Michanthidium albitarse* **22** *Michanthidium almeidai* sp. n. **23–24** male genitalia in dorsal view **23** *Michanthidium almeidai* sp. n. **24** *Michanthidium albitarse*. Scale line= 1.0 mm (Figure 18). Scale line = 0.25 mm (Figures 19–20; 23–24). Scale line = 0.5 mm (Figures 21–22).

#### Etymology.

The specific epithet is homage to our friend and bee specialist, Dr. Eduardo Andrade Botelho de Almeida, professor at the Universidade de São Paulo, Ribeirão Preto (FFCLRP).

#### Comments.

The paratype male from Bahia State is probably a teneral specimen judging the pale coloration of legs and sterna. The paratype from Sergipe State has the sterna black with light yellow bands and tibiae and femora with black areas. The wings and antennae are partially damaged.

##### Key to *Michanthidium* species

**Table:** 

1	Male	2
–	Female	4
2(1)	Head and mesosoma without ferruginous areas; sixth tergum with a large translucent apical margin, angulated at middle (Fig. 22); seventh tergum with wide rounded lateral lobes, without median spine and with a rounded median depression (Fig. 20)	*Michanthidium almeidai* sp. n.
–	Head and mesosoma with ferruginous areas; sixth tergum with straight apical margin (Fig. 21); seventh tergum with lateral lobes acute, with or without median spine and without a median depression (Fig. 19)	3
3(2)	Hind coxa with midapical spine; sixth tergum with small sublateral tooth; seventh tergum without median spine (Fig. 19)	*Michanthidium albitarse*
–	Hind coxa without midapical spine, with a short row of black, thick short hairs on median margin; sixth tergum without sublateral tooth; seventh tergum with median spine	*Michanthidium ferrugineum*
4(1)	Lateral margin of axilla broadly rounded; posterior margin of scutellum subtruncate and laterally expanded in a large translucent area (Fig. 12); external surface of tibiae and basitarsi with small, fine punctures, and pilosity decumbent; shorter than ocellar diameter; tegula densely punctate, punctures separated by one or less puncture diameter	5
–	Lateral margin of axilla subangulate; posterior margin of scutellum weakly emarginated at middle, laterally expanded in a small translucent area near axillae (Fig. 11); external surface of tibiae and basitarsi with coarser punctures, pilosity longer than ocellar diameter; tegula sparsely punctate, punctures separated by much more than one puncture diameter	*Michanthidium almeidai* sp. n.
5(4)	Head mostly black with limited ferruginous areas (Fig. 3); mesoscutum laterally with coarse, large punctures (about one-third median ocellar diameter); distal tergum not medially projecting into spine	*Michanthidium albitarse*
–	Head almost totally ferruginous (Fig. 2); mesoscutum uniformly punctate; punctures small (about one-fourth to one-fifth median ocellar diameter); distal tergum medially projecting into spine (Fig. 16)	*Michanthidium ferrugineum*

## Discussion

The curved hairs on the underside of the labial palpi are shared with *Larocanthidium* Urban, 1997, although in *Michanthidium* those hairs are also present throughout the length of the galeal blade in both sexes. This morphology suggests a special floral relationship (Michener 2007) although there is scarce information about the plants visited by *Michanthidium* species. Gonzalez and Griswold (2011) reported that the examined females and males of *Michanthidium ferrugineum* were collected on flowers of *Cuphea* sp. (Lythraceae). The holotype female of *Michanthidium almeidai* sp. n. was collected on *Scoparia dulcis* Linnaeus (Scrophulariaceae) and the paratype male from Canindé de São Francisco on *Schultesia guianensis* (Aublet) Malme (Gentianaceae). Although these are only isolated records, they could be a first step to understanding specialization in these bees.

## Supplementary Material

XML Treatment for Michanthidium almeidai
